# Effect of clear aligner type on maxillary full-arch intrusion: 3D analysis using finite element method

**DOI:** 10.1186/s12903-024-03984-6

**Published:** 2024-02-13

**Authors:** Allahyar Geramy, Fatemeh Safari

**Affiliations:** Department of Orthodontics, School of Dentistry, Tehran University of Medical Sciences, Tehran, Iran

**Keywords:** Clear aligners, Tooth intrusion, Finite element analysis

## Abstract

**Background:**

Vertical maxillary excess (VME) is one of the most common reasons for seeking orthodontic treatment. Total intrusion with aligners is a promising alternative to surgery in some cases. Considering the elastic deformation of aligners, this study aimed to evaluate the possible desirable and undesirable teeth displacements during full maxillary arch intrusion using clear aligners and temporary anchorage devices (TADs).

**Methods:**

The maxillary arch and clear aligners were modeled in SolidWorks. Four aligner brands including Leon, Duran, Duran Plus, and Essix Plus were selected based on their material properties. Anterior and posterior intrusion forces of 80 and 300 g were applied from attachments between the canines and first premolars and between the first and second molars, respectively. Vertical and anteroposterior tooth displacements were determined.

**Results:**

The greatest intrusion was recorded at the buccal of the second molar, followed by the first molar. The lowest value was measured at the palatal of the molars with all aligners except Duran, which indicated minimal intrusion in the central incisor. All teeth were mesially displaced at the incisal/occlusal except incisors that moved distally. All apices showed distal movement.

**Conclusions:**

Total intrusion using clear aligners may be accompanied by other tooth movements, including buccal tipping and mesial-in rotation of the molars, retrusion of incisors, and mesial movement of other teeth.

## Background

Vertical maxillary excess (VME), also known as gumminess is an esthetic issue that negatively impacts patients’ self-esteem and psychological well-being. It is defined as the display of more than 3 mm of gingiva at smile [1–3]. VME is the second most common reason for seeking treatment, with a prevalence of approximately 22% among the Asian population [4, 5]. This condition occurs due to excessive vertical growth of the maxilla and a recommended treatment option for adults is a Lefort I osteotomy, which involves superior repositioning both the anterior and posterior segments of the maxilla [6]. However, many patients are reluctant to undergo surgery due to the associated risks and potential complications [7]. Fortunately, LeFort I impaction can be mimicked to some extent by total maxillary arch intrusion using Temporary Anchorage Devices (TADs) [8, 9]. Intrusive force can be applied by attaching elastics from both the fixed orthodontic appliances’ wire and clear aligners to the TADs.

Clear aligners have become a popular alternative to traditional fixed appliances, particularly for orthodontic patients seeking more esthetic treatments [10]. They offer advantages in terms of both the esthetic treatment process and quality of life when compared to traditional fixed appliances [11–13]. Since their introduction by Kesling in 1946, clear aligners have undergone significant development and have been increasingly used over the past two decades to treat various degrees of malocclusion [14–18]. Due to their coverage of the entire dentition, aligners may provide effective teeth intrusion and a more stable anchorage unit [19–21].

Previous studies, have explored the application of intrusive force using clear aligners to address different malocclusions. For example, Harris et al. evaluated the correction of anterior openbite by analysing cephalometric superimpositions in patients treated with clear aligners. They observed that maxillary and mandibular molar intrusion, mandibular autorotation, and incisor extrusion contributed to the closure of the openbite [22]. Liu et al. investigated the intrusion of anterior teeth in the treatment of deep bite cases using clear aligners. They discovered that different intrusion set ups with the same activation exerted varying forces on incisors, canines, and premolars [23]. In another cases, Lin et al. successfully treated a patient with bimaxillary protrusion and a gummy smile by extracting four premolars and utilizing clear aligners and TADs. Elastic attachments to miniscrews allowed for buccal segment retraction and anterior segment intrusion, ultimately achieving ideal overbite and overjet and correcting the gummy smile [24].

Despite these previous studies, there is still limited research on the potential unwanted tooth movements that may occur during full-arch intrusion using clear aligners. Therefore, the main objective of this study was to evaluate teeth movement in the sagittal and horizontal planes during full-arch maxillary intrusion with clear aligners and TADs.

## Methods

### Preparation of models and material properties

This study was conducted at the Department of Orthodontics, Tehran University of Medical Sciences. To assess the intrusion of the entire maxillary arch, models of the maxillary arch and four common clear aligners with two attachments in each quadrant were created. Intrusion forces were applied from the attachments to the miniscrews placed distal to the canine and distal to the first molar.

The maxillary arch, which consists of cortical and spongy bones, teeth, and PDLs was modeled using SolidWorks 2015 (SolidWorks, Concord, MA, USA) based on Wheeler’s dental anatomy [25]. All materials were set up as isotropic and homogenous [26]. The PDL was designed with a uniform thickness of 0.25 mm. The mechanical properties of the biological structures are presented in Table 1. The maxillary model was the same for all types of aligners.

**Table 1:** Mechanical properties of teeth, PDL, and bones

	Young’s modulus (MPa)	Poisson’s ratio
Tooth	20,300	0.26
PDL	0.667	0.49
Cancellous bone	13,400	0.38
Cortical bone	34,000	0.26

PDL: Periodontal ligament

**Fig. 1:**
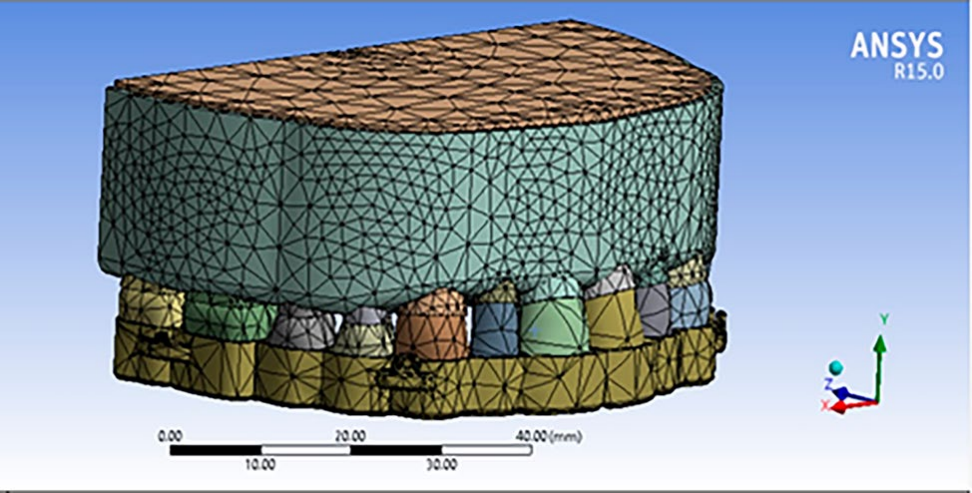
Maxillary arch with clear aligner model

Four commercially available clear aligners with different material properties include Leon (St. Louis Park, MN, USA), Duran (Scheu-Dental, GmbH, Iserlohn, Germany), Duran Plus (Scheu Dental GmbH, Iserlohn, Germany), and Essix Plus (Dentsply Sirona Deutschland, GmbH, Bensheim, Germany) were designed with thicknesses of 0.8 mm, 1 mm, 0.75 mm, and 0.9 mm, respectively, according to their mechanical characteristics (Table 2), [27, 28]. A Poisson’s ratio of 0.36 was established for the aligners based on previous studies [29, 30]. They were considered isotropic and homogeneous materials [26]. Two attachments in each quadrant, located at the midpoint between the canine and first premolar; and at the midpoint between the first and second molars, were designed to serve as hooks for applying intrusion force (Fig. 1). The intrusive forces were applied at designated locations in the aligners. Miniscrews were not designed to reduce the model size.

**Table 2:** Mechanical properties of different aligners

	Thickness (mm)	Young’s modulus (MPa)	Poisson’s ratio
Duran Plus®	0.75	2746	0.36
Leon	0.8	1239.5	0.36
Essix® PLUS™	0.90	1869	0.36
Duran	1.0	2227	0.36

### Boundary conditions and loading

The models were meshed in Ansys Workbench Version 15 (Ansys Inc., Southpointe, Canonsburg, PA, USA). Bonded contacts were defined between the tooth-PDL, PDL-cancellous bone, and PDL-cortical bone to simulate the biological situation. The same contacts were established between the teeth and the aligners based on the direction of loading. A boundary condition was applied to limit the displacement of nodes at the superior and posterior surfaces of the cortical and spongy bones, as well as mesiodistal aspect of the model in all three-dimensional planes. Intrusion forces of 80 and 300 g were applied from the attachments in each quadrant between the canine and first premolar and between the first and second molars, respectively. The software determined the vertical and anteroposterior displacements of the teeth at incisal/occlusal and apical arbitrary points.

## Results

**Fig. 2:**
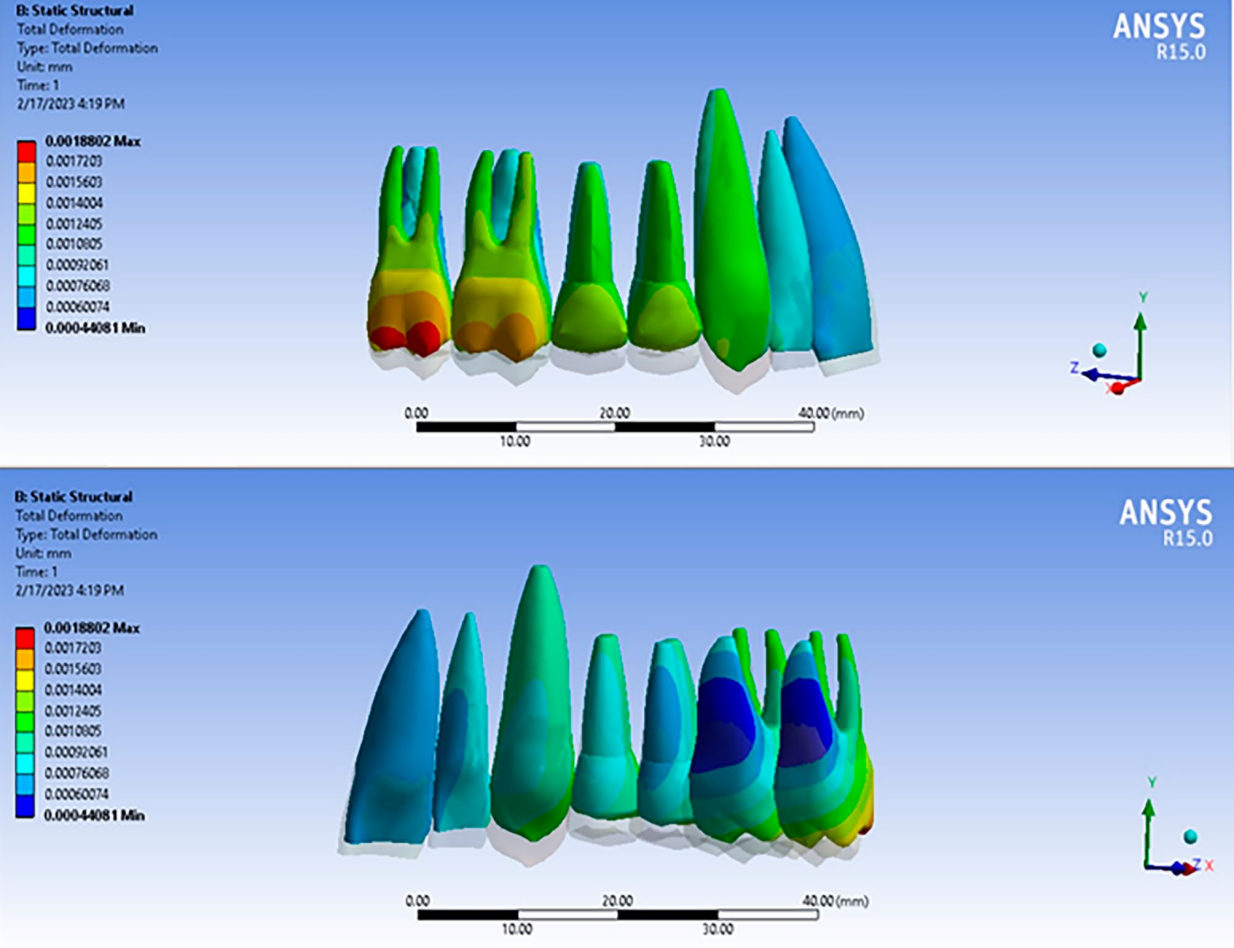
Simulation example showing vertical displacement of teeth in buccal and palatal views

**Fig. 3:**
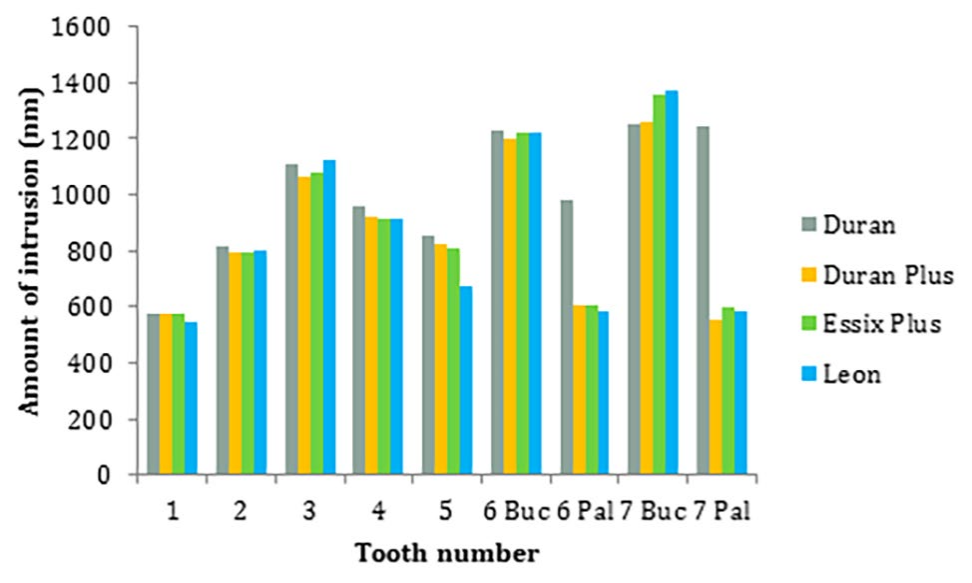
Intrusion of teeth with different aligners including Duran, Duran Plus, Essix Plus, and Leon. Pal: Palatal / Buc: Buccal

Vertical displacement with different aligners indicated a similar trend (Figs. 2 and 3). The highest intrusion was recorded at the buccal point of the second molar by 1370, 1358, 1259, and 1252 nm with Leon, Essix plus, Duran plus, and Duran, respectively. The second highest intrusion values were observed at the buccal point of the first molar by 1227, 1220, 1219, and 1203 nm with Duran, Essix plus, Leon, and Duran plus, respectively. The third highest intrusions were indicated at the canine by 1121, 1121, 1076, and 1063 nm with Leon, Duran, Essix plus, and Duran plus, respectively. The least amount of intrusions with Leon, Duran plus, and Essix plus were shown at the central incisors by 544, 574, and 578 nm, respectively. Then the lowest values were recorded at the palatal point of the second molar followed by the palatal point of the first molar for Leon, Duran plus, and Essix plus. The minimal amount of intrusion with Duran was recorded at the central incisor followed by the lateral incisor and second premolar.

**Fig. 4:**
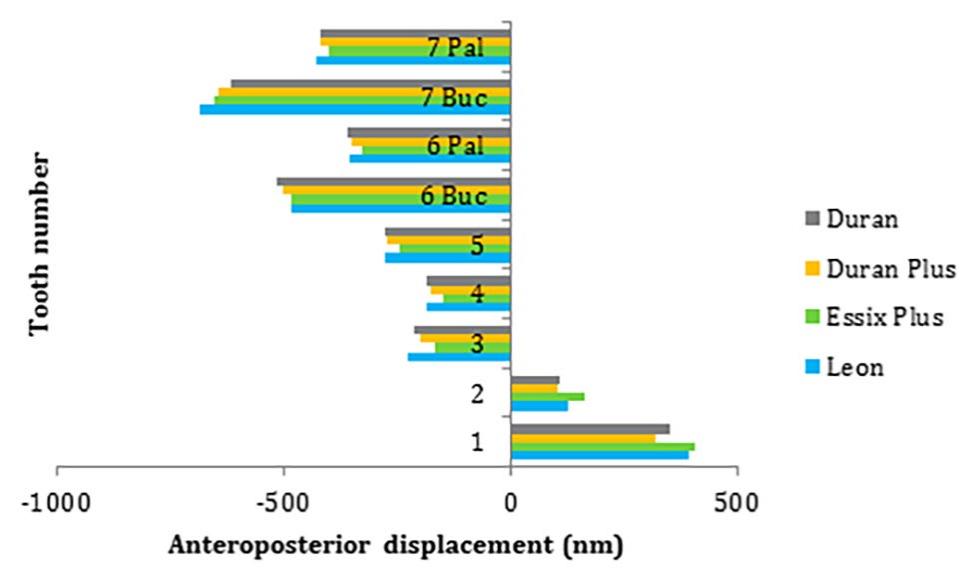
Anteroposterior displacement of teeth with different aligners including Duran, Duran Plus, Essix Plus, and Leon at incisal/occlusal points. Pal: Palatal / Buc: Buccal/ Positive values: Distal movement/ Negative values: Mesial movement

Anteroposterior measurements at incisal/occlusal points showed the distal movement of the incisors and mesial movement of other teeth (Fig. 4). The greatest distal movement occurred at the central incisor with Essix plus, Leon, Duran, and Duran plus by 407, 393, 351, and 319 nm, respectively. The greatest mesial movement was recorded at the buccal point of the second molar with Leon, Duran plus, Duran, and Essix plus by 426, 421, 419, and 401 nm, respectively which was followed by the buccal point of the first molar, palatal point of the second molar, palatal point of the first molar, second premolar, canine, and first premolar.

**Fig. 5:**
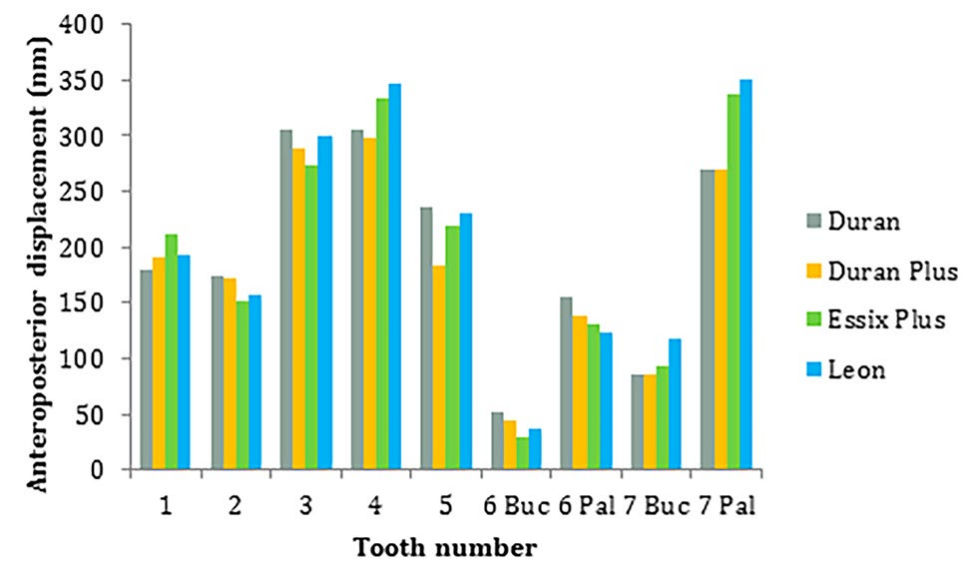
Anteroposterior displacement of teeth with different aligners including Duran, Duran Plus, Essix Plus, and Leon at the apical points in nm. Pal: Palatal / Buc: Buccal

Anteroposteriorly, all apices moved distally (Fig. 5). The greatest amount of apical movement with Leon was recorded at the palatal point of the second molar and first premolar by 350 nm and 347 nm, respectively. Duran showed the greatest apical movement by 306 nm at both canine and first premolar. Essix plus and Duran plus resulted in the greatest apical movement at the first premolar by 333 and 298 nm, respectively. The lowest apical movement with all aligners was observed at the buccal point of the first molar ranging from 29 to 52 nm.

## Discussion

Clear aligners are increasingly used to address various malocclusions. This treatment modality is compatible with teeth intrusion due to the whole dentition coverage and exertion of light forces [19, 20, 31]. The thermoplastic material composition offers the advantage of light force generation to aligners, but can also cause aligner deformation, affecting tooth movements and treatment results [31, 32]. In the present study, the trends of tooth movements were taken into account rather than the exact values. The amount of tooth movement and its clinical significance require further assessment.

Poisson’s ratio plays an important role in statics analysis. In this study, the Poisson’s ratios for all materials were considered to be 0.36. This means that the tension on an aligner causes it to lengthen by 0.36 in the direction of tension and contract by 0.36, perpendicular to the tension. Poisson’s ratio did not affect the results of this study, as the values were the same for all aligners.

Based on the results of this study, the amount of intrusion with all aligners except Duran was significantly different between the buccal and the palatal sides of molars (Fig. 3). Greater intrusion was observed at the buccal region which may result in buccal tipping of the molars. The buccal tipping of the molars during intrusion may be explained by the expected moment. Aligners are able to apply force from all directions due to engagement with the occlusal, buccal, and lingual teeth surfaces. This property is referred to as “the watermelon seed effect”. It may be supposed that the resultant force directed through the tooth center of resistance [33, 34]. However, the resultant force mostly does not pass through the center of resistance and creates a moment. This is due to the uneven force distribution caused by the non-symmetrical structure of the tooth crown [35]. To counteract this moment and redirect the force through the center of resistance, it has been proposed to add “pressure areas” to the aligner [35, 36]. Additionally, buccal tipping can be controlled to some extent by adding palatal attachments. Increasing the number of attachments has been suggested to reduce aligner deformation [30].

In this study, the difference between the buccal and palatal intrusion of molars was smaller with Duran aligner compared to other aligners. This may be attributed to the effect of thickness and elastic modulus on elastic deformation [37, 38]. Modulus of elasticity is an important physical characteristic that indicates the magnitude of force of thermoforming materials. The low modulus of elasticity of clear aligners (nearly 40–50 times lower than NiTi archwires) makes them susceptible to deformation even at low forces [35]. Thicker sheets may be suggested to compensate for the low modulus of elasticity. In this study, Duran aligner was the thickest and had the second highest modulus of elasticity. The combination of these two characteristics in Duran aligner appears to have reduced the tendency for buccal tipping of the molars.

Among anterior teeth, the amount of intrusion increased from the central incisor to the canine. This may be explained by the location of the miniscrew and force application. Consistent with the present study, Geramy et al. investigated maxillary full arch intrusion with Duran clear aligners and various miniscrew placements. They found that incisor intrusion was greater than canine intrusion when the miniscrews were distal to the lateral incisor and distal to the first molar, whereas canine intrusion was greater than incisor intrusion when the anterior miniscrew was located distal to the canine, as in this study [39].

In the sagittal plane, lingual movement of incisors and mesial movement of other teeth occurred at the incisal/occlusal points. This is expected in a clinical setting, as the aligners must be stretched to fit the dentition. Therefore, aligners may exert a retracting force on the anterior teeth and a protracting force on the posterior teeth. Patients are often instructed to place the aligners on their front teeth first and then on their posterior teeth, which may cause the entire aligner to move back. Thus, posterior protraction can be more significant [40]. Intraoral elastics may be recommended to reinforce the anchorage.

According to the results, the apices of the incisors moved lingually, similar to their crowns. However, the lingual movement of the central incisor was greater at the incisal tip than at the apex for all aligners. Therefore, incorporation of power ridges may be considered to control the torque. The amount of lingual movement of the lateral incisor was almost similar at the incisal and apical points. Consistent with this finding, Jiang et al. reported greater lingual root movement with increasing intrusion movement. This tendency decreased from the central incisors to the lateral incisors [41]. This may be due to the different positions of central and lateral incisors in the dental arch, and the different crown morphologies.

The apices of the canine, premolars, and molars moved distally, in the opposite direction to the crown movement. This may suggest mesial tipping of the crowns. Similarly, Fan et al. revealed mesial tipping of the second molar irrespective of attachment position due to the counterclockwise moment generated during molar intrusion [30]. The prescription can be modified by adding an additional distal crown tip on the posterior teeth to prevent mesial tipping. In addition, molars mesial movements were greater at the buccal than the palatal aspect. Therefore, one caution during the intrusion by aligners may be the mesial-in rotation of the molars. These observations were also possibly due to the bowing effect reported by Zhu et al. in a micro-sensor study [42]. Fan et al. believe that the bowing effect is unavoidable [30]. However, this phenomenon does not necessarily occur in clinical settings [43–45].

The clinical efficacy of clear aligners may be influenced by various factors. Elastic deformation is a significant factor in treatment outcomes [32]. Previous studies have also emphasized the impact of thermoforming and the oral environment on the aligners’ properties. Thermoforming resulted in changes in the transparency, thickness, hardness, and water solubility of the aligners [46, 47]. Variations in aligner thickness and hardness have also been observed in studies simulating the oral environment [48, 49], although Bucci et al. showed that the thickness change will not adversely affect clinical performance [47]. Lombardo et al. indicated that the force exerted by aligners is affected by the oral environment [50]. In contrast, Elshazley et al. showed that the effect of saliva on applied force and torque was insignificant [51]. Given the controversy in the literature and the limitations of simulation studies, it seems logical to expect variability in estimated tooth movement in a clinical setting. This should be considered in order to draw conclusions. Thus further studies, including clinical investigations, are warranted to confirm the findings of the current study.

Orthodontic models prototyped with 3D printing technologies including stereolithography (SLA), digital light processing (DLP), and liquid crystal display (LCD) have been shown to be accurate and can be used as a diagnostic tool or for indirect production of clear aligners [52]. LCD printers may offer a promising low budget alternative to professional 3D printers with a clinically acceptable inaccuracy below 0.25 mm [53, 54]. Future studies evaluating the possible teeth movements during total maxillary arch intrusion comparing different 3D manufacturing processes are recommended.

## Conclusions

Within the limitations of this study, total intrusion of the maxillary arch may be accompanied by other undesirable tooth movements such as buccal tipping of the molars, retrusion of incisors, mesial migration of other teeth, and mesial-in rotation of the molars.

## Data Availability

All data generated or analysed during this study are included in this published article.

## Abbreviations

FEM: Finite Element Method
VME: Vertical Maxillary Excess
TAD: Temporary Anchorage Device
